# Influenza Transmission in a Community during a Seasonal Influenza A(H3N2) Outbreak (2010–2011) in Mongolia: A Community-Based Prospective Cohort Study

**DOI:** 10.1371/journal.pone.0033046

**Published:** 2012-03-13

**Authors:** Nao Nukiwa-Souma, Alexanderyn Burmaa, Taro Kamigaki, Ishiin Od, Namuutsetsegiin Bayasgalan, Badarchiin Darmaa, Akira Suzuki, Pagbajabyn Nymadawa, Hitoshi Oshitani

**Affiliations:** 1 Department of Virology, Tohoku University Graduate School of Medicine, Sendai, Japan; 2 National Influenza Center, National Center of Communicable Diseases, Ulaanbaatar, Mongolia; 3 Baganuur District, Ulaanbaatar, Mongolia; 4 Mongolian Academy of Medical Sciences, Ulaanbaatar, Mongolia; University of Hong Kong, Hong Kong

## Abstract

**Background:**

Knowledge of how influenza viruses spread in a community is important for planning and implementation of effective interventions, including social distancing measures. Households and schools are implicated as the major sites for influenza virus transmission. However, the overall picture of community transmission is not well defined during actual outbreaks. We conducted a community-based prospective cohort study to describe the transmission characteristics of influenza in Mongolia.

**Methods and Findings:**

A total of 5,655 residents in 1,343 households were included in this cohort study. An active search for cases of influenza-like illness (ILI) was performed between October 2010 and April 2011. Data collected during a community outbreak of influenza A(H3N2) were analyzed. Total 282 ILI cases occurred during this period, and 73% of the subjects were aged <15 years. The highest attack rate (20.4%) was in those aged 1–4 years, whereas the attack rate in those aged 5–9 years was 10.8%. Fifty-one secondary cases occurred among 900 household contacts from 43 households (43 index cases), giving an overall crude household secondary attack rate (SAR) of 5.7%. SAR was significantly higher in younger household contacts (relative risk for those aged <1 year: 9.90, 1–4 years: 5.59, and 5–9 years: 6.43). We analyzed the transmission patterns among households and a community and repeated transmissions were detected between households, preschools, and schools. Children aged 1–4 years played an important role in influenza transmission in households and in the community at large. Working-age adults were also a source of influenza in households, whereas elderly cases (aged ≥65 years) had no link with household transmission.

**Conclusions:**

Repeated transmissions between households, preschools, and schools were observed during an influenza A(H3N2) outbreak period in Mongolia, where subjects aged 1–4 years played an important role in influenza transmission.

## Introduction

Influenza is a relatively mild disease and most of its cases are self-limiting. However, it can cause serious complications such as pneumonia, especially in the elderly and young children [1]. Influenza also causes pandemics such as that in 2009 [2], which can lead to high morbidity and mortality worldwide. Various social distancing measures have been considered for mitigating the impact of influenza pandemics, including, household isolation, quarantine, and school closures [3]–[6]. It is important to know how influenza viruses spread in a community for planning and implementation of effective social distancing measures. Households are believed to be a major site of influenza virus transmission and many studies have been conducted on household transmission to define the transmission characteristics of seasonal and pandemic influenza viruses [7]–[12]. Children have a higher attack rate of influenza than adults, and schools are also believed to be an important site of influenza transmission. Children infected in schools may become a source of subsequent transmission to households and communities [13]–[15]. This is why school closures can be effective in reducing the impact of influenza on a community [5], [16]–[18]. Households and schools are known to have an important role in influenza transmission in a community, but the overall picture of community transmission has not been defined for actual outbreaks, including the exact proportions of household and school transmission and their interactions. A prospective cohort study is a more appropriate method for analyzing influenza transmission in a community [19]–[21]. However, such studies are resource-intensive and rarely conducted.

Mongolia is a landlocked country with the second lowest population density in the world (1.74/km^2^ in 2010). Despite this low population density, influenza transmission occurs almost every year [22]. We previously reported that there was a significant burden of influenza during the influenza season, and the incidence of influenza-like illness (ILI) was particularly high in children aged <5 years [23]. Based on this result, we hypothesized that influenza transmission was driven by preschool children in Mongolia. Our previous study also indicated that the incidence of ILI was low among adults, but this might be more attributable to their health-seeking behavior, because adult patients with ILI are less likely to visit healthcare facilities than children [24]. And this may have led to an underestimation of the true incidence of ILI in adults. Therefore, we conducted a community-based cohort study instead of a healthcare facility-based study to capture the overall transmission patterns in a community. The objective of this study was to describe the transmission characteristics of influenza in a community in order to capture the true incidence of influenza in different age groups and to determine the roles of households, schools, and preschools in influenza transmission in a local Mongolian community.

## Methods

The study was conducted in Baganuur District, Ulaanbaatar City, which is located 130 km east of the center of Ulaanbaatar City. The main industry in this area is coal mining. The population of Baganuur District was 26,905 in 2010, and the average annual population growth rate was 1.3% (2006–2010). Forty percent of the residents lived in apartments and the rest in traditional houses known as ger or private houses. Unlike in rural areas of Mongolia, most gers and private houses in Baganuur District are built close together, forming a community. There was 1 general hospital and 4 family general practices (FGP) in this district. The FGP is an outpatient clinic that patients visit first when they are sick, and residents usually visit a designated FGP. There are generally 3–4 doctors and 4–5 nurses in each FGP, and patients are referred to the general hospital if they have a severe condition that requires hospitalization. We conducted a prospective cohort study among the residents of an area covered by one FGP.

The baseline information for the study population was collected between July and September 2010. The baseline information included the address, contact number, size and structure of households, demographic information on each resident, and their affiliation. The study was performed between October 2010 and April 2011. Trained nurses contacted each household at least once a week by telephone to check whether there had been any ILI cases in the household during the previous week. Investigators visited the households in presence of an ILI case in the house and interviewed the residents after obtaining written informed consent. An adult member was interviewed as a proxy if the ILI subject was unable to answer questions or they were under 18 years of age, after obtaining written informed consent. Information regarding symptoms, the date of onset, and household contact information was collected by means of a standardized questionnaire. Nasopharyngeal or oropharyngeal swabs were collected from all ILI patients in whom the onset of symptoms had occurred within 7 days. The samples were transferred to the National Influenza Center in Ulaanbaatar City, where they were tested to detect the influenza virus using real-time reverse transcriptase-polymerase chain reaction (RT-PCR), according to the protocol provided by the Centers for Disease Control and Prevention in the United States of America [25]. Specific primers and probes were used for influenza A(H3N2), A(H1N1)pdm09, and B viruses. A portion of the RNA extract was transferred and re-tested by real-time RT-PCR at Tohoku University Graduate School of Medicine, Japan using previously described methods [26]. An individual was considered to be influenza positive when influenza virus was detected by at least 1 of the 2 laboratories. Influenza positive rate for each day was calculated as the number of influenza positive samples divided by the number of samples tested.

ILI was defined as fever (≥38°C) or feverishness with a cough and/or a sore throat. An index case was defined as anyone who had ILI when there were no ILI cases in the household during the previous 7 days before the onset of their symptoms. A household contact was defined as any person who had stayed in the same household with the index case for at least 2 nights from 1 day before to 7 days after the onset of the index case. Since most of the secondary cases are known to occur within 7 days after the illness onset of index cases [27], a secondary case was defined as a household contact who developed an ILI 1 to 7 days after the onset of the index case. Distinctions were not made between secondary and tertiary cases in the household. Household transmission was considered to have occurred if at least 1 household contact became a secondary case. Household contacts that did not develop an ILI within 7 days after the onset of the index case were classified as uninfected household contacts. In this analysis, when an ILI case occurred among the household contacts within 7 days after the illness onset of index case, this case was assumed to acquire infection in the household and was considered as “household transmission secondary case” and the index case as “household transmission index case”. The remaining index cases that did not have ILI cases among household contacts within 7 days after their onsets were considered as “non-household transmission ILI cases”. “Household transmission index case” and “non-household transmission ILI case” were assumed to have acquired infection outside the households, including schools and preschools. The crude household secondary attack rate (SAR) was calculated as the number of secondary cases divided by the total number of eligible household contacts. Cases which involved households with only 1 member were excluded from the analysis. The serial interval was believed to be >1 day [28]; hence, when 2 index cases had the same onset date, the infection was considered to have a different source outside of the household.

Two two-dimensional contour maps were created using Matlab (MathWorks, MA, USA) to visualize how transmissibility progressed over time in the community by showing the density plot of ILI cases. One was a density plot of “household transmission index case” and “non-household transmission ILI case”. The other was a density plot of “household transmission secondary case”. Epidemic curves of each schools and preschools were described to observe the outbreaks.

Proportions and distributions were compared using the chi-square test or Fisher's exact test. Mean values were compared using the Student *t*-test or Mann-Whitney U test. Statistical significance was assessed using two-tailed tests with an error level of 0.05. The statistical analysis was conducted using SPSS Statistics version 18 (IBM, IL, USA). This study was approved by the Research Ethics Committees of the Tohoku University Graduate School of Medicine, Sendai, Japan and the Mongolian Academy of Medical Sciences, Ulaanbaatar, Mongolia.

## Results

### Characteristics of the study population

There were 1,417 households with 5,887 residents in the study area and among them, 6 households (34 residents) refused to participate and 68 households (198 residents) were excluded because of not possessing the contact number. Therefore, a total of 5,655 residents (96.1%) in 1,343 households (94.8%) were included in this cohort study. Among them, 1,268 households (94.4%) occupied apartments, 40 households (3%) occupied traditional houses (ger), and 35 households (2.6%) occupied private houses. The demographic characteristics of the study population are summarized in Table 1. The median age of the subjects was 25 years (range, 0–94 years). The age distribution of the study population was similar to that of the national population, but the proportion aged 1–4 years was higher in the study population. The male to female ratio was 0.99. The median household size was 4 persons (range, 1–13 persons). In the study population, 404 subjects (7.1%) attended 5 preschools, while 1,134 (20.1%) attended 4 schools, all of which were located in the study area.

**Table 1:** Demographic characteristics of total study population and all ILI cases during the overall study period, ILI cases and influenza A(H3N2) positive cases during influenza A(H3N2) outbreak period, and household transmission index cases and household transmission secondary cases.

		Total study population (n = 5655)	All ILI cases (n = 708)	ILI cases during influenza A(H3N2) outbreak period (n = 282)	Influenza A(H3N2) positive cases (n = 79)	Household transmission index cases (n = 43)	Household transmission secondary cases (n = 51)
Gender	Male	2814 (49.8%)	346 (48.9%)	138 (48.9%)	39 (49.4%)	19 (44.2%)	25(49.0%)
	Female	2841 (50.2%)	362 (51.1%)	144 (51.1%)	40 (50.6%)	24 (55.8%)	26 (51.0%)
	Total	5655 (100%)	708 (100%)	282 (100%)	79 (100%)	43 (100%)	51 (100%)
Age	<1 y	146 (2.6%)	67 (9.5%)	18 (6.4%)	2 (2.5%)	2 (4.7%)	5 (9.8%)
	1–4 y	510 (9.0%)	252 (35.6%)	104 (36.9%)	27 (34.2%)	20 (46.5%)	12 (23.5%)
	5–9 y	444 (7.9%)	93 (13.1%)	48 (17.0%)	16 (20.3%)	6 (14.0%)	14 (27.5%)
	10–14 y	502 (8.9%)	92 (13.0%)	36 (12.8%)	11 (13.9%)	5 (11.6%)	6 (11.8%)
	15–19 y	573 (10.1%)	43 (6.1%)	12 (4.3%)	2 (2.5%)	1 (2.3%)	3 (5.9%)
	20–29 y	1094 (19.3%)	39 (5.5%)	21 (7.4%)	6 (7.6%)	4 (9.3%)	6 (11.8%)
	30–39 y	821 (14.5%)	36 (5.1%)	9 (3.2%)	5 (6.3%)	3 (7.0%)	1 (2.0%)
	40–49 y	740 (13.1%)	34 (4.8%)	16 (5.7%)	5 (6.3%)	2 (4.7%)	3 (5.9%)
	50–64 y	567 (10.0%)	32 (4.5%)	9 (3.2%)	3 (3.8%)	0 (0%)	1 (2.0%)
	≥65 y	258 (4.6%)	20 (2.8%)	9 (3.2%)	2 (2.5%)	0 (0%)	0 (0%)
	Total	5655 (100%)	708 (100%)	282 (100%)	79 (100%)	43 (100%)	51 (100%)
	Mean age ± SD (years)	27.90±19.12	14.09±18.35	13.72±17.62	14.96±17.29	10.58±12.86	12.27±13.65
	Median age (years)	25	6	6	7	4	7
	Range (years)	0–94	0–94	0–80	0–70	0–47	0–62

Abbreviations: ILI, influenza-like illness; SD, standard deviation.

### Characteristics of ILI cases during the overall study period

**Figure 1:**
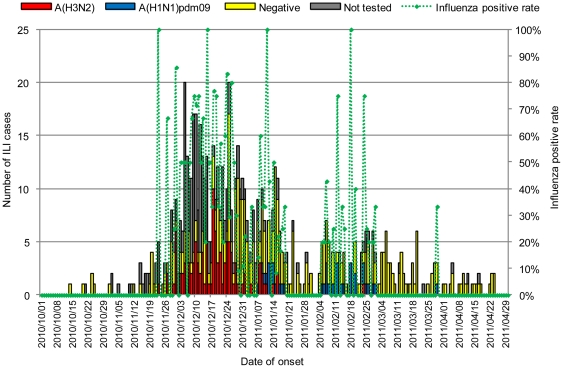
Epidemic curve of ILI cases and the proportion influenza-positive samples. Influenza A(H3N2) positive cases are shown in red, influenza A(H1N1)pdm09 positive cases are shown in blue, influenza negative ILI cases are shown in yellow, and ILI cases without sample collection are shown in gray. Influenza positive rate for each day was calculated as the number of influenza positive samples divided by the number of samples tested.

There were a total of 708 ILI cases during the study period. The greatest influenza activity was seen between December 2010 and January 2011 (Figure 1). The demographic characteristics of all ILI cases are shown in Table 1. The median age of all ILI cases was 6 years (range, 0–94 years) and children aged 1–4 years accounted for 35.6% of cases. Seven of the ILI patients were hospitalized and discharged without sequelae. Of the 708 ILI cases, samples from 501 cases (70.8%) were collected and 384 (76.6%) of them were collected within 3 days of their onset of illness. Influenza A(H3N2) virus was detected in 108 samples (21.6%) and influenza A(H1N1)pdm09 virus in 37 samples (7.4%), whereas there were no influenza B-positive samples. Influenza A(H3N2) virus was the predominant strain in December 2010, whereas a mixture of influenza A(H3N2) and A(H1N1)pdm09 viruses was detected in January 2011. The proportion of samples positive for influenza decreased after December 25 (Figure 1). This might be due to other respiratory pathogens that were circulating in this area during the time of study. To further analyze influenza transmission in the community, we only focused on periods when influenza A(H3N2) virus was dominant, and when the influenza positive rate was high, i.e., from November 23 to December 24, 2010. During this influenza A(H3N2) virus outbreak period, 282 ILI cases occurred. Samples were collected from 143 ILI cases, and influenza A(H3N2) virus was detected in 79 cases (55.2%). The demographic characteristics of the 79 influenza A(H3N2) virus-positive cases and the remaining influenza A(H3N2) virus-negative ILI cases or ILI cases with no sample collection were similar (Table 1); therefore, we focused on these 282 ILI cases for further analysis based on the assumption that most of these ILI cases during this period were infected by influenza A(H3N2) virus. The majority of ILI cases (206/282 = 73%) occurred in those aged <15 years, while more than one-third of the cases occurred in those aged 1–4 years.

### Household transmission during the influenza A(H3N2) outbreak period

**Figure 2:**
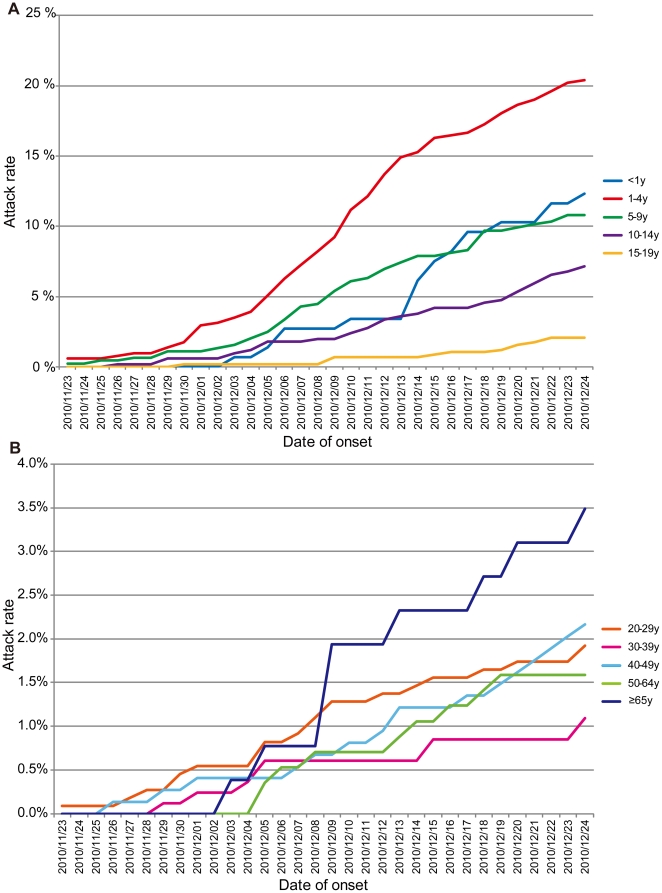
Attack rate during the influenza A(H3N2) outbreak period. Attack rate for (A) children aged <20 years and (B) adults aged ≥20 years.

Figure 2 shows the attack rates for each age group during the influenza A(H3N2) outbreak period. The highest attack rate during this period was observed among children aged 1–4 years (20.4%) (Figure 2A). In contrast, the final attack rate for those aged 5–9 years was 10.8%. The attack rate for those aged <1 year increased gradually with a sudden increase after December 13, and their final attack rate was 12.3%. The final attack rates for adults was lower than that for children, but an increase in those in their 20 s and 40 s was observed earlier than in other adult age groups. The number of ILI cases (n = 9) was less, but the highest final attack rate (3.5%) among adult age groups was found in those aged ≥65 years and an increase was observed in this age group after December 8 (Figure 2B).

A total of 43 households experienced secondary case(s) during this period. There were 36 households in which one secondary case occurred, 6 households in which 2 secondary cases occurred, and 1 household in which 3 secondary cases occurred. An analysis based on the size of the household indicated that the secondary attack rate was higher in household containing ≥4 persons, while households containing 5 persons had significantly higher SAR (Table 2). Fifty-one secondary cases occurred among 900 household contacts to give an overall crude household SAR of 5.7%. We calculated the SAR based on the age groups and gender of the household contacts to determine the risk of transmission among household contacts. The SAR was significantly higher when the household contacts were <10 years, whereas it was highest among those aged <1 year (Table 3). There was no significant difference between genders. We also assessed the risk of influenza transmission based on the age groups of the index cases. In contrast to the age of household contacts, there were no significant associations between the age groups of the index cases and SAR (Table 4). Of the 51 secondary cases, 24 cases (47.1%) had index cases aged 1–4 years. In contrast, only 2 cases had index cases aged <1 year. There were no secondary cases in those aged ≥5 years for index cases aged <1 year. Most of the secondary cases from index cases aged 20–49 years occurred in those aged <20 years. No secondary cases occurred for index cases aged ≥50 years (Table 4).

**Table 2:** Secondary attack rates and relative risk of ILI by household size.

Household size	No. of households	No. of households with secondary cases	SAR	Relative risk (95% CI)
≤3 persons	42	3	7.1%	1.00 (Reference)
4 persons	71	13	18.3%	2.56 (0.78–8.48)
5 persons	57	15	26.3%	3.68 (1.14–11.92)
≥6 persons	62	12	19.4%	2.71 (0.81–9.02)
Total	232	43	18.5%	

Abbreviations: ILI, influenza-like illness; SAR, secondary attack rate; CI confidence interval.

**Table 3:** Secondary attack rates and relative risk of ILI by age and gender of household contacts.

		No. of household contacts	No. of secondary cases	SAR	Relative risk (95%CI)
Age	<1 y	16	5	31.3%	9.90 (3.39–28.90)
	1–4 y	68	12	17.6%	5.59 (2.18–14.31)
	5–9 y	69	14	20.3%	6.43 (2.57–16.06)
	10–14 y	70	6	8.6%	2.71 (0.91–8.14)
	15–19 y	83	3	3.6%	1.14 (0.29–4.47)
	20–29 y	190	6	3.2%	1.00 (Reference)
	30–39 y	178	1	0.6%	0.18 (0.02–1.46)
	40–49 y	102	3	2.9%	0.93 (0.24–3.65)
	50–64 y	78	1	1.3%	0.41 (0.05–3.32)
	≥65 y	46	0	0.0%	NA
Gender	Male	472	25	5.3%	1.00 (Reference)
	Female	428	26	6.1%	1.15 (0.67–1.96)
Total		900	51	5.7%	

Abbreviations: ILI, influenza-like illness; SAR, secondary attack rate; CI, confidence interval; NA, not applicable.

**Table 4:** Secondary attack rates and relative risk of ILI by age group of index cases.

Age group of index cases	No. of index case	Age group of household contact	No. of household contacts (%)	No. of secondary cases (%)	SAR	Relative risk (95%CI)
<1 y	13	<1 y	1 (2%)	0 (0%)	0.0%	
		1–4 y	4 (9%)	2 (100%)	50.0%	
		5–19 y	10 (22%)	0 (0%)	0.0%	
		20–49 y	24 (52%)	0 (0%)	0.0%	
		≥50 y	7 (15%)	0 (0%)	0.0%	
		Subtotal	46 (100%)	2 (100%)	4.3%	1.00 (0.23–4.30)
1–4 y	91	<1 y	6 (2%)	3 (13%)	50.0%	
		1–4 y	21 (6%)	4 (17%)	19.0%	
		5–19 y	86 (23%)	10 (42%)	11.6%	
		20–49 y	215 (58%)	7 (29%)	3.3%	
		≥50 y	44 (12%)	0 (0%)	0.0%	
		Subtotal	372 (100%)	24 (100%)	6.5%	1.49 (0.77–2.87)
5–19 y	75	<1 y	6 (2%)	2 (15%)	33.3%	
		1–4 y	24 (8%)	1 (8%)	4.2%	
		5–19 y	76 (25%)	7 (54%)	9.2%	
		20–49 y	154 (51%)	2 (15%)	1.3%	
		≥50 y	40 (13%)	1 (8%)	2.5%	
		Subtotal	300 (100%)	13 (100%)	4.3%	1.00 (Reference)
20–49 y	37	<1 y	2 (1%)	0 (0%)	0.0%	
		1–4 y	18 (13%)	5 (42%)	27.8%	
		5–19 y	42 (30%)	6 (50%)	14.3%	
		20–49 y	56 (41%)	1 (8%)	1.8%	
		≥50 y	20 (14%)	0 (0%)	0.0%	
		Subtotal	138 (100%)	12 (100%)	8.7%	2.01 (0.94–4.28)
≥50 y	16	<1 y	1 (2%)	0	0.0%	
		1–4 y	1 (2%)	0	0.0%	
		5–19 y	8 (18%)	0	0.0%	
		20–49 y	21 (48%)	0	0.0%	
		≥50 y	13 (30%)	0	0.0%	
		Subtotal	44 (100%)	0	0.0%	NA
All age	232	Total	900	51	5.7%	

Abbreviations: ILI, influenza-like illness; SAR, secondary attack rate; CI, confidence interval; NA, not applicable.

**Figure 3:**
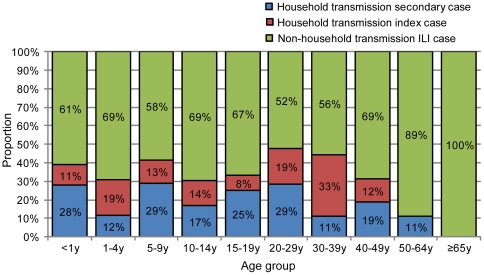
Proportion of household transmission index cases, household transmission secondary cases, and non-household transmission ILI cases. Household transmission secondary cases were assumed to be transmitted in households. Household transmission index cases and non-household transmission ILI cases were assumed to be transmitted outside households.

**Figure 4:**
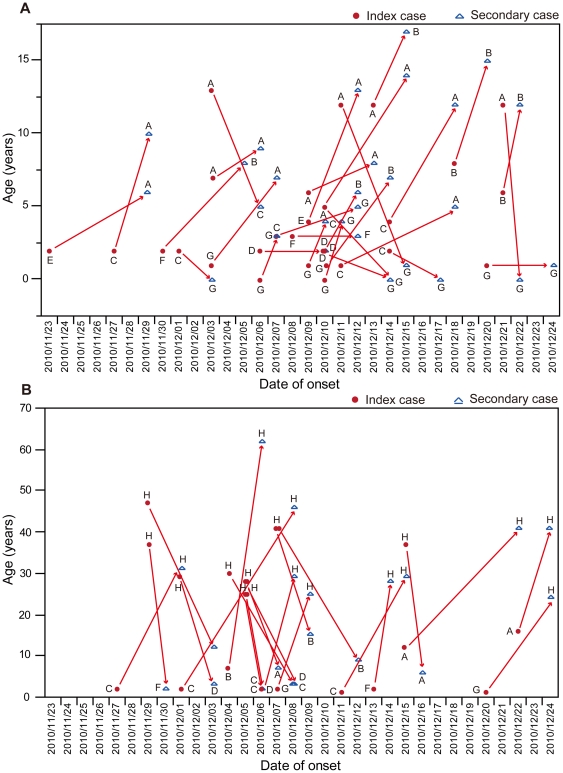
Relationship between index cases and secondary cases with household transmission. Relationship of household transmission (A) among children and (B) among children and adults. Red circles represent index cases, blue triangles represent secondary cases, and arrows show the direction of transmission. The letters represent affiliations; A: school A; B: school B; C: preschool C; D: preschool D; E: preschool E; F: preschool F; G: children at home; H: adults.

Figure 3 shows the proportion of 3 ILI groups for each age group. The proportions of “household transmission index case” and “non-household transmission ILI case” that were assumed to have acquired infection outside the households were 88% for those aged 1–4 years, 89% for those aged 30–39 years and 50–64 years, and 100% for those aged ≥65 years. However, the proportions of “household transmission secondary case” that were assumed to have acquired infection in households were 28% for those aged <1 year, 29% for those aged 5–9 years and 20–29 years. Figure 4 shows the relationship between the household transmission index and secondary cases based on the date of the onset. Twenty-nine (56.9%) household transmissions occurred between children, and 19 (65.5%) of these were from younger to older children, 7 (24.1%) from older to younger children, and 3 (10.3%) among same age children (Figure 4A, Table S1). A further 21 (41.2%) household transmissions occurred between children and adults. Of these, 8 (38.1%) were from children to their parents (6 to the mother and 2 to the father/uncle), 11 (52.4%) were from parents to children (8 from the mother and 3 from the father), and 2 (9.5%) were from children to grandmothers (Figure 4B, Table S1). Only 1 household transmission occurred between adults (between brothers). These results suggest that the occurrence of transmission among children aged 1–4 years was greatest in the community and this was most likely to have occurred in preschools, whereas children aged 5–9 years were more likely to have acquired influenza in households, mainly from their younger siblings. Working-age adults also might have introduced influenza into the household, and mothers were more commonly involved in household transmission than fathers.

### Transmission between households and the community

**Figure 5:**
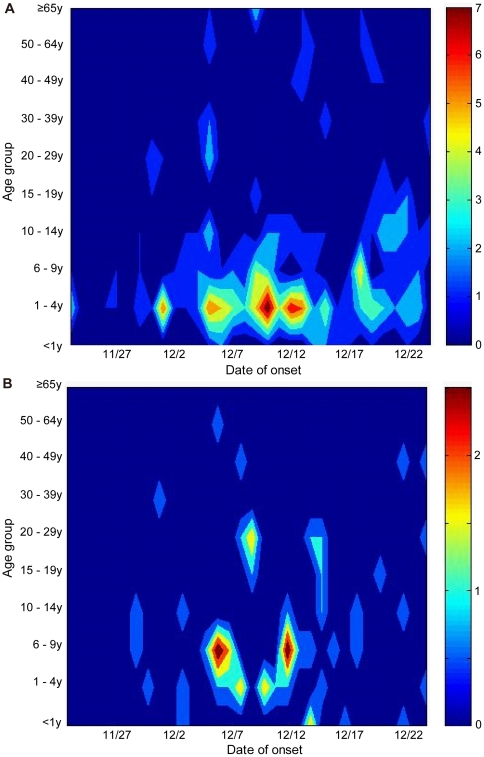
Density plot on the basis of age group and date of onset. Density plot of (A) household transmission index cases and non-household transmission ILI cases (n = 231) and (B) household transmission secondary cases (n = 51).

**Figure 6:**
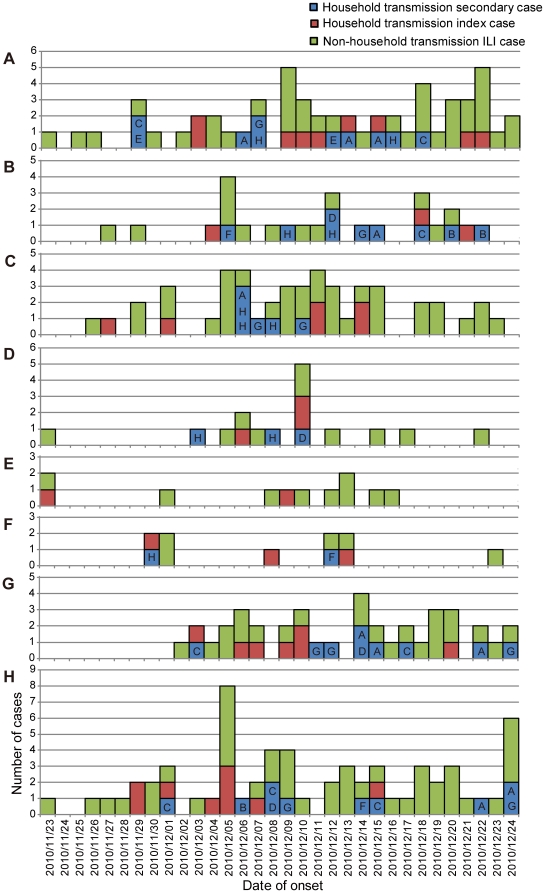
Epidemic curves for each affiliation. Epidemic curves of household transmission index cases, household transmission secondary cases, and non-household transmission ILI cases in (A) school A, (B) school B, (C) preschool C, (D) preschool D, (E) preschool E, (F) preschool F, (G) children at home, and (H) adults. The letters attributed to the household transmission secondary cases are the affiliations of their index cases; A, school A; B, school B; C, preschool C; D, preschool D; E, preschool E; F, preschool F; G, children at home; H, adults.

We analyzed the transmission patterns between households and the community. Figure 5 shows the density plot of ILI cases based on the age group and date of the onset. The density plot of “household transmission index cases” and “non-household transmission ILI cases” shows the distribution of ILI cases that were likely to have acquired infections in the community (Figure 5A). In contrast, the density plot of “household transmission secondary cases” shows the distribution of ILI cases that were likely to have acquired infections in households (Figure 5B). There were 4 schools and 5 preschools in the study area and Table 5 shows the distribution of study population and the number of ILI cases occurred for each affiliation. The overall attack rate in preschools was significantly higher than that in schools, i.e., 84/404 (20.8%) vs. 82/1,134 (7.2%) (*p*<0.01). Information on children who stayed at home and adults was also included in Table 5. We constructed epidemic curves based on these affiliations in order to analyze the interaction between household transmission and influenza outbreaks in schools and preschools (Figure 6). ILI cases in this epidemic curve were divided into 3 groups; “household transmission index case”, “household transmission secondary case”, and “non-household transmission ILI case”. The affiliations of “household transmission index cases” were indicated in the epidemic curves for “household transmission secondary cases”, i.e., 2 schools (A, B), 4 preschools (C, D, E, F), children who stayed at home (G), and adults (H) (Figure 6). Two schools and 1 preschool that had <10 children had only 1 case of ILI (Table 5), therefore, these small schools and preschools were not included in Figure 6. Our density plot analysis shows that high density (i.e., clusters of cases) occurred among those aged 1–4 years several times during the influenza A(H3N2) outbreak period (Figure 5A). Peaks in the epidemic curves of preschools were synchronized with these high densities (Figure 6), indicating that ILI cases in this age group was likely to have occurred in preschools. Sporadic cases were noted in schools (A and B), preschools (C, D, and E), and adults (H) between November 23 and 28, and household transmission started to occur sporadically after November 29. It should be noted that no ILI cases occurred among children at home (G) until December 2 (Figure 6). There were several possible household transmissions from adults to children immediately after the highest number of adult cases (H) was observed on December 5 (Figure 4B, Figure 6). Schools (A) and preschools (C and D) had an increasing number of cases after infection by these adults (Figure 5A, Figure 6). After the outbreak in preschools (C and D), it appeared that some preschool children transmitted infection to their school-aged siblings in households. These secondary cases appeared to result in school outbreaks (A and B) (Figure 5A, Figure 6). Most of the secondary cases in older school-aged children (10–14 years) occurred after December 12. These results indicated that outbreaks started in preschools and that there were subsequent household transmission from preschool children to school-aged children. These results suggested that there were repeated transmissions between households, preschools, and schools. Adults who were infected outside of households were also thought to become a source for the introduction of the virus into households.

**Table 5:** Distribution of study population, ILI cases, secondary cases, and ILI attack rates for each affiliation.

	Affiliation		Study population (median age)	No. of ILI cases	No. of secondary cases (%)	ILI attack rate
1	School	A	787 (12 y)	56	10 (18%)	7.1%
2	School	B	337 (10 y)	25	9 (36%)	7.4%
3	School	-	8 (12 y)	1	1 (100%)	12.5%
4	School	-	2 (7.5 y)	0	NA	0%
5	Preschool	C	240 (3 y)	47	6 (13%)	19.6%
6	Preschool	D	68 (3 y)	16	3 (19%)	23.5%
7	Preschool	E	58 (3 y)	11	0 (0%)	19.0%
8	Preschool	F	32 (3 y)	10	2 (20%)	31.3%
9	Preschool	-	6 (3.5 y)	0	NA	0.0%
10	Children at home	G	304 (1 y)	39	9 (23%)	12.8%
11	Adults	H	3206 (39 y)	62	10 (16%)	1.9%

Abbreviations: ILI, influenza-like illness; NA, not applicable.

### Transmission in elderly residents

There were only 9 ILI cases in those aged ≥65 years, and their attack rate increased slowly compared with younger adults, reaching a final attack rate of 3.5% (Figure 2B). None of them were “household transmission index case” or “household transmission secondary case” (Figure 3), indicating that their infections had probably occurred outside of household. The mean size of the households where these elderly residents lived was 2.7, which was significantly lower than that of the other age groups (*p*<0.05). Three elderly ILI cases lived only with their partners (also aged ≥65 years) and only 1 household with an elderly ILI case had school-aged children. These profiles indicated that residents aged ≥65 years had a low probability of contact with children in their households and a higher probability of acquiring influenza in the community after the outbreak expanded.

## Discussion

We analyzed the incidence of ILI in households and the temporal distribution patterns between households, schools, and preschools during an influenza A(H3N2) outbreak period in a local Mongolian community. The ILI attack rate was highest among children aged 1–4 years, and this rate was almost the double of that for children aged 5–9 years. A similar age distribution of influenza cases was also observed in our previous study where we analyzed surveillance data from seasonal and pandemic influenza outbreaks [23], and a serological study of pandemic A(H1N1)2009 virus in Mongolia [29]. The high ILI attack rate noted among small children might be due to their susceptibility to influenza A(H3N2) virus, because there had been no major outbreak of influenza A(H3N2) virus since 2005/2006 season in Mongolia and the seasonal influenza vaccination was not routinely provided. A higher attack rate among small children with influenza A(H3N2) virus compared with A(H1N1) virus has also been reported from other countries [19], [30]. In Mongolia, both parents work in many households and many children aged ≥2 years attend preschools, and some preschools (e.g., preschool C) have relatively greater numbers of children. In countries like Mongolia, preschools may be a major focus of influenza transmission. The attack rate was low, but we observed ILI cases in adults, even in those aged ≥65 years. These adult cases were underreported by healthcare facility-based surveillance [23], probably because adults are less likely to visit healthcare facilities due to influenza infections compared with children. Data from sentinel surveillance is insufficient for determining the actual burden of influenza and analyzing how influenza is transmitted in the community, whereas community-based studies with active case-finding are more suitable for capturing the overall transmission patterns in a community [24]. Although our study site is a small community of 1,417 households with 5,887 residents, majority of households participated in the study and only 74 households (5.2%) with 232 residents (3.9%) were not included in the study. Therefore we believe that overall transmission patterns in the community were adequately captured.

The overall crude household SAR was 5.7% in our study. Previous studies of household transmission have reported an overall SAR of 5%–24% for seasonal influenza [28], [31]–[33] and 4%–26% for pandemic A(H1N1)2009 [11], [12], [27], [34], [35]. It is difficult to compare this study with previous studies because the study designs are different. SAR may depend on the influenza subtypes or strains, the susceptibility of household contacts, or any interventions provided to the household contacts including antiviral prophylaxis and non-pharmaceutical intervention. In many studies of household transmission, the laboratory confirmed index cases were recruited by healthcare facilities and their household contacts were followed up [36]. In such a study design, the health-seeking behavior of the population also affects the SAR. In this study, a higher SAR was observed in younger household contacts, and half of the household transmission occurred between children in the same household. This is possibly because of susceptibility, and the contact patterns in children that have greater opportunity for transmission. This result was compatible with previous studies of seasonal influenza and pandemic A(H1N1)2009 [28], [37], [38]. However, there was no significant association between the age of index cases and SAR. This contrasted with a previous study conducted in France on influenza A(H3N2) [28]. Mothers were involved in two-thirds of the household transmission between children and parents. Another household transmission study conducted on pandemic A(H1N1)2009 also found that mothers had a higher SAR because they were attentive exclusively to the index cases [27], [39].

Temporal expansion of influenza in the households and community indicated that there were repeated transmissions between households, schools, and preschools. Our data also suggested that those aged 1–4 years had an important role in household and community transmissions. Strong inter-site interactions with back-and-forth waves of possible transmission between schools, community, and households were also detected during the pandemic in 2009 [40]. However, school-aged children (aged 6–18 years) facilitated the introduction and spread of influenza in households during that study. Our study also found that adults might have introduced influenza into households and transmitted it to their children. It was not clear how the influenza A(H3N2) virus was first introduced into this community. Sporadic cases were seen during the early stage of the outbreak (i.e., mid November), and the early increase in adult cases (aged 20–29 years and 40–49 years) suggested that the initial introduction might have been facilitated by adults. It should be noted that many young adults, such as university students, frequently travel between Baganuur District and center of Ulaanbaatar City. There was a cluster of adult cases on December 5 and some of them became index cases of household transmission. It appeared that children infected by adults in households might have spread the virus via their schools and preschools. Physicians in Mongolia usually recommend children to stay at home during the first 3–5 days after their onset of ILI. However, children tend to go back to schools and preschools after having no fever or 2–3 days after their onset of symptoms. Most of the elderly people in our study population lived separately from their children and grandchildren, and they appeared to have a low probability of contact with young children, which probably made their SAR low. The slow increase in the attack rate of the elderly suggested that they were infected after influenza had broadly expanded in the community.

There are several limitations in our study. First, we could not provide sufficient sample collection kits at the start of the study, and the proportion of ILI cases with samples was low until mid-December. Therefore, we defined the influenza A(H3N2) outbreak period when the proportion of samples positive for influenza A(H3N2) virus was high enough to assume that most ILI cases during this period were affected by this virus, even though there were ILI cases with no sample collected or influenza-negative samples. However, this may mean that we might have overestimated the overall attack rate in this analysis. Second, we only focused on the influenza A(H3N2) outbreak period, which was the largest outbreak during the 2010/2011 season. The subtypes of influenza might influence the transmission patterns in a community, but we only observed a small outbreak of influenza A(H1N1)pdm09 virus in January 2011. This study should be repeated in subsequent influenza seasons to reveal the transmission patterns of different subtypes and strains in Mongolia. Third, we identified ILI cases by contacting households and inquiring whether any household member was presenting ILI. Therefore, we could not detect any asymptomatic cases that might be involved in household transmission. A serological study conducted during the pandemic period reported that an estimated 9.4% of influenza A(H1N1)pdm09 virus infections acquired in households were asymptomatic [41]. We used ILI case definition which includes fever or feverishness as inclusion criteria. Therefore, we might have also missed to include influenza cases with no fever episode. Review of volunteer challenge studies conducted in the past showed only 34.9% of influenza positive cases had fever (≥37.8°C) [42]. It is also possible that some ILI cases with short duration of illness were missed by active case-finding using the telephone since we only called each household once a week. Fourth, we assumed that all secondary cases were acquired by transmission in households even though we only observed temporal distribution of ILI cases. In reality, it is not possible to differentiate whether the transmission of secondary cases occurred in households or in the community [43]. Longini et al. [7] produced a mathematical model to help avoid this problem. And recent studies using viral genetic sequence showed that viruses isolated from the same household were usually derived from the same viral lineage and this method will make it possible to differentiate where secondary cases acquired infections [41], [44]. Fifth, we did not conduct contact tracing for each case. Therefore, we could not know whether the study subjects actually attended school or preschool while they still had infectivity or if there were any contacts between cases at schools and preschools. Finally, we produced an epidemic curve for schools and preschools based on the data for study population and not for the whole school or preschool population. Therefore, the actual timing of outbreaks in schools and preschools might be different.

Despite these limitations, we were able to describe how influenza transmission occurred in a Mongolian community. Awareness of seasonal and pandemic influenza has improved in developing countries over the past decade because of the threat of a highly pathogenic avian influenza A(H5N1) virus and influenza A(H1N1)pdm09 virus. However, epidemiological data on influenza in developing countries is still very limited. The epidemiology of influenza may vary between countries, because the transmission patterns in a community are likely to be influenced by various demographic and social factors such as age structure, household size, and social mixing patterns. It is necessary to understand the epidemiology of influenza in different social contexts so that feasible and effective control strategies can be developed in resource-limited settings. Our study is the first study of the community transmission of influenza in Mongolia and one of the few studies conducted in resource-limited settings. A prospective cohort study on influenza, such as the present study, can provide data on the transmission dynamics of influenza in a community. However, such studies are rarely conducted because of high costs and methodological issues. At our study site, the FGP doctors were trusted by and had a close relationship with most of the residents, which facilitated frequent follow-ups and sample collection. Transmission studies in such a close community can provide useful data for understanding influenza transmission.

## Supporting Information

Table S1 **Relationship between index cases and secondary cases of household transmission.**
(TIF)
